# Tip60‐dependent acetylation of KDM2B promotes osteosarcoma carcinogenesis

**DOI:** 10.1111/jcmm.14497

**Published:** 2019-06-20

**Authors:** Xin Shi, Mingfu Fan

**Affiliations:** ^1^ Department of Spinal Surgery Linyi People's Hospital, Beicheng New District Hospital Linyi China

**Keywords:** acetylation, KDM2B, nucleosomes, osteosarcoma, Tip60

## Abstract

Overexpression of KDM2B is frequently occurred in various human solid tumours, and the high levels of KDM2B are associated with tumourigenesis. However, whether and how its activities might be modulated to facilitate tumour progression is still unclear. Immunoprecipitation and immunoblotting were carried out to detect the acetylation of KDM2B. Nucleosomes and mononucleosomes were prepared and the demethylation activity of KDM2B was detected in these two substrates. The effects of KDM2B acetylation on the transcription of target genes, as well as tumour growth and metastasis were then studied. KDM2B was acetylated in osteosarcoma cancer cell lines (MG‐63 and HOS). This modification occurred at lysine 758 and catalysed by Tip60. Acetylation of KDM2B decreased the capacity of KDM2B in binding with nucleosomes. KDM2B acetylation diminished its demethylation activity towards nucleosomal substrates rather than towards bulk histone. Besides, acetylation of KDM2B diminished its ability to bind with the promoters of *p21* and *puma*. Moreover, the promoting effects of KDM2B acetylation on tumour cells' proliferation and metastasis, and in vivo tumour growth were dependent on Tip60. KDM2B is acetylated at lysine 758 by Tip60 in human osteosarcoma cells. Acetylation of KDM2B diminishes its association with nucleosomes, and thus increasing methylation of H3K36 at its target genes as well as enhancing its oncogenic effects.

**Highlights**

- KDM2B is acetylated in osteosarcoma cancer cells;
- KDM2B acetylation occurs at lysine 758 and catalysed by Tip60;
- KDM2B acetylation diminishes its demethylation activity towards nucleosomes;
- KDM2B acetylation diminishes its ability to bind with *p21* and *puma*;
- Tip60‐dependent KDM2B acetylation exerts oncogenic effects.

## 1 INTRODUCTION

Osteosarcoma is the most frequent primary solid malignancy of bone in the young adolescent.^1^ Histopathologically, this cancer is defined by the presence of spindle‐shaped malignant mesenchymal cells which produce abnormal osteoid and/or immature bone, and featured by rapid local invasion and early pulmonary metastases. Unlike other sarcomas genetically featured by specific chromosomal translocation, osteosarcoma is hallmarked by genomic instability including high degree of aneuploidy, the aggregation of unbalanced chromosomal rearrangements and multi‐regional amplifications and deletions.^2^ Due to poor response to a variety of chemotherapy, the therapeutic improvement on osteosarcoma was very modest during the past two decades.^3^ At present, the overall 5‐year survival rate of patients with localized osteosarcoma was about 70%‐80%, while it falls to 20%‐30% in patients with metastases.^4^ In addition, the prognosis for patients with non‐metastatic osteosarcoma who had local recurrence remains quite dismal with 13% long‐term survival.^5^ A better understanding of osteosarcoma is required which will be important for developing novel therapeutic targets.

In eukaryote, genomic DNA is packaged in chromatin by twining with histone octamer. Chromatin can be covalently modified, such as DNA methylation and histone modification. The amino terminus of histone subunits is called histone tails free, and many residues on histone tails can be covalently modified. Different modifications of different residues can induce a variety of different signals, which can regulate the expression of genes in eukaryotes, and thus produce a variety of biological effects. Studies have shown that histone modifications are closely related to the pathogenesis of many diseases, such as retinopathy,^6^ psoriasis 7 and various cancers.^8^ In the field of osteosarcoma, histone modification has been linked with the onset and progression of this tumour. For instance, histone deacetylase (HDAC) 2 was reported to play significant regulatory roles in osteosarcoma cancer stem cells phenotype and in vivo cancer growth.^9^ Likewise, Jiang et al^10^ demonstrated that, histone methyltransferase SETD2 inhibited osteosarcoma cells growth and chemosensitivity.

Generally, the modifications of histone include phosphorylation, methylation, ubiquitination and acetylation, among which histone methylation plays significant roles in the process of post‐transcriptional regulation. Histone methylation often occurs at lysine (K) and arginine (R) residues, and is mediated by both histone methyltransferases and histone demethylases. To date, several histone lysine demethylases have been identified, including KDM1, KDM2 (A, B), KDM3 (A, B), KDM4 (A, B, C, D), KDM5 (A, B, C, D) and KDM6 (A, B). KDM2B, also known as JHDM1B/FBXL10/NDY1, acts only on mono‐ and dimethylated H3K36. Recently, KDM2B has drawn a great deal of attention, since its role in regulating tumourigenesis via epigenetic mechanisms.^11^, ^12^ In vitro and in vivo data showed that, KDM2B up‐regulated the expression of histone methyltransferase EZH2 to promote the progression of ovarian cancer.^13^ In mouse model of pancreatic cancer, silence of KDM2B abrogated tumourigenicity of pancreatic ductal adenocarcinoma cells.^14^

Depending on different structural homologies and biochemical mechanisms, acetylases can be classified into five groups, including GNATs family (Hat1, Ccn5, PCAF, Elp3, Hpa2), MYST family (Esa1, Morf, Ybp2, Sas2, Sas3, Tip60, Hbo1), P300/CBP family, nuclear receptor co‐activators (SRC‐1, TIF2, ACTR), as well as TAFII250 and TFIIIC family (TFIIIC220, TFIIIC110, TFIIIC90). Tip60 was the first human MYST family member that exhibits acetyltransferase activity towards both histone and non‐histone proteins.^15^, ^16^ It is one of the well‐known lysine acetyltransferase, which has been reported to be involved in tumourigenesis of diverse human cancers, like colon, breast and prostate tumours.^17^

In the present study, we investigated if KDM2B acetylation could be involved in the onset and progression of osteosarcoma. Moreover, whether Tip60 is responsible for the acetylation of KDM2B in osteosarcoma cells was studied.

## 2 MATERIALS AND METHODS

### 2.1 Cell lines and treatment

Human osteosarcoma cell lines MG‐63 (CRL‐1427™) and HOS (CRL‐1543™), as well as a human embryonic kidney cell line HEK293 (CRL‐1573™) were purchased from ATCC (Manassas, VA). These cell lines were all cultured in Eagle's Minimum Essential Medium (ATCC) supplemented with 10% foetal bovine serum (Gibco, Grand Island, NY). All cells were maintained at 37°C in a humid incubator with 5% CO_2_.

Vetec™ reagent grade of Trichostatin A (TSA) and Nicotinamide (NAM) with purity of ≥98% purchased from Sigma (St. Louis, MO) were respectively used as inhibitors of HDAC. MG‐63 and HOS cells were treated with 80 nmol/L TSA^18^ or 5 mmol/L NAM^19^ for 24 hours.

### 2.2 Antibodies

Anti‐acetyl‐lysine (Ac‐K; ab21623), anti‐Tip60 (ab137518), anti‐Myc (ab9106), anti‐p53 (ab26), anti‐p21 (ab188224), anti‐puma (ab9643), anti‐HA (ab18181) and anti‐KDM2B (ab137547) antibodies were purchased from Abcam (Cambridge, MA). Anti‐H3K36me2 (#2901) and anti‐H3K36me3 (#4909S) antibodies were purchased from Cell Signaling Technology (Danvers, MA). The antibody specific for Flag (F7425) was purchased from Sigma (St. Louis, MO).

### 2.3 Plasmids, transfection and qRT‐PCR

The 3'UTR regions of wild‐type (WT) of KDM2B and Tip60 were amplified by PCR and the PCR products were inserted into Flag‐, HA‐ or Myc‐tagged vectors (EK‐Bioscience, Shanghai, China). The empty vectors were used as blank controls. The KDM2B^K758R^ and KDM2B^K758Q^ constructs were mutated using site‐directed mutagenesis. A shRNA specific for Tip60 was inserted into a cloning vector of shRNA pLKO.1‐puro (Sigma).

For qRT‐PCR, the total RNAs were extracted by Trizol reagent (Invitrogen, Carlsbad, CA). Reverse transcription and qPCR were carried out by commercial kits, that is, Transcriptor First Strand cDNA Synthesis Kit and FastStart Universal SYBR Green Master (both from Roche, Basel, Switzerland). Data were calculated by 2^−ΔΔCt^ method and were normalized against β‐actin.

### 2.4 Co‐immunoprecipitation and chromatin immunoprecipitation

Cells were cross‐linked with 1% formaldehyde for 10 minutes. After crosslinking, the cells were washed twice with PBS. Cell pellets were collected and further washed with washing buffer (0.25% TritonX‐100, 10 mnol/L EDTA, 0.5 mmol/L EGTA, 10 mmol/L Tris, pH 8.0), and re‐suspended in sonication buffer (1 mmol/L EDTA, 0.5 mmol/L EGTA and 10 mmol/L Tris, pH 8.0), mixed with glass beads and then subjected to sonication process. Immunoprecipitation was carried out by using antibodies against KDM2B, H3K36me2, H4K16ac and Flag. The immunoprecipitates were subjected to a series of washing steps to remove non‐specific binding materials. The DNA samples were purified and analysed by qRT‐PCR.

### 2.5 Purification of recombinant protein from bacteria

Flag‐tagged KDM2B (aa 1‐851) and its mutants (K‐R and K‐Q), as well as Tip60 (aa 1‐458) were generated by PCR, cloned into N‐terminal 6 × His‐tag bacterial expression vector pET30a (Novagen, Madison, WI). The constructs were verified by DNA sequencing. The plasmids were transformed into bacteria and the expression of the target proteins was induced by 0.1 mmol/L isopropyl‐β‐D‐thiogalactoside (IPTG; Solarbio, Beijing, China). The tagged proteins were purified by Ni‐NTA affinity column (Qiagen, Hilden, Germany). Thereafter, the column was washed, and the elute was dialysed as previously described.^20^ The concentration of proteins was analysed on SDS‐PAGE by Coomassie blue staining using BSA as standard control.

### 2.6 In vitro acetylation assay

After the Flag‐Tip60 and Flag‐KDM2B proteins were precipitated by anti‐Flag antibody from HEK293 cells, the precipitates were incubated with recombinant KDM2B or its mutated types. The acetylation reactions were mediated by the following reagents: 50 mmol/L Tris (pH 8.0), 10% glycerol, 1 mmol/L DTT, 10 mmol/L sodium butyrate, 20 μmol/L of acetyl‐CoA, control beads or Flag‐Tip60 immunoprecipitates and 100 ng of recombinant His‐tagged‐KDM2B full‐length or Tower domain (WT or K‐R mutant) purified from bacteria. The reaction mixture was loaded on SDS‐PAGE gels, followed by immunoblotting.

### 2.7 Demethylase assay and nucleosome binding assay

To obtain oligonucleosomes, a nuclear pellet from approximately 1 × 10^8^ cells Hela cells was treated with lysis buffer (20 nmol/L HEPES, PH7.5; 0.25 mol/L sucrose; 3 mmol/L MgCl_2_; 0.5% NP40; 3 mmol/L 2‐mercaptoethanol). The oligonucleosomes were then digested with micrococcal nuclease (10 units/mL) and mononucleosomes were derived as previously described.^21^ KDM2B demethylation activity assay on free bulk histones or nucleosomal histones were carried out as previously reported.^21^

### 2.8 Cell viability assay

After the indicated transfection, HOS cells (2 × 10^4^) were seeded in 60 mm plates for 5 days. The cell growth curve was draw by counting living cells.

The viability of MG‐63 cells after transfection was detected by using CCK‐8 reagent (Dojindo Molecular Technologies, Kyushu, Japan). The OD values of each sample was detected by an ELISA reader (Bio‐Rad Laboratories) for evaluating cell viability.

### 2.9 Colony formation assay

The transfected HOS cells (500 cells/well) in culture medium were mixed with 0.35% low‐melting agarose. The mixture was placed in 6‐well plates which were pre‐coated with 0.6% low‐melting agarose. After 2 weeks of incubation, the number of colonies was counted.

### 2.10 Ki‐67 staining

The proliferation of HOS cells was tested by Ki‐67 Cell Proliferation Kit (Sangon Biotech, Shanghai, China). In short, cells were fixed with 4% paraformaldehyde and incubated with 0.1% Triton X‐100. After blocking with 3% BSA, the samples were probed by anti‐Ki67 rabbit antibody (dillution 1:100). Fifty‐microlitres of Cy3‐conjugated goat anti‐rabbit IgG (dillution 1:100) was then added. The Ki‐67 stained cells were counted under fluorescence microscope.

### 2.11 BrdU assay

The proliferative capacity of MG‐63 cells after the indicated transfection was also determined by Bromodeoxyuridine (BrdU) assay kit (Roche). The rate of BrdU‐positive cells were calculated by counting the stained cells under fluorescence microscope.

### 2.12 Wound closure

5 × 10^5^ transfected HOS cells were seeded in 6‐well plates and the cultures were incubated at 37°C for 12 hours. Wounds were made by 200 μL pipette tips. After washing by PBS for three times, serum‐free medium was added and the cultures were incubated for 24 hours. The area of the closed wound was calculated by using NIH ImageJ imaging software (National Institutes of Health, Bethesda, MD).

### 2.13 Invasion assay

The invasive capacities of HOS and MG‐63 cells were measured by a 24‐well transwell chamber with 8‐μm pore insert (Corning, NY). The transfected cells were seeded in the upper side of the chamber with serum‐free medium. The lower chamber was filled with the complete culture medium. 24 hours later, the cells in the lower side were stained by crystal violet and counted directly.

### 2.14 Mouse tumour models

A total of 96 SPF grade NOD/SCID mice (5‐6 weeks old) were purchased from Vital River Laboratories (Beijing, China). Animal experiments of this study were approved by the Animal Ethics Committee of Linyi People's Hospital, and all procedures were performed in accordance with the ethical standards. Mice were randomly divided into four groups (24 mice per group), namely: vector, WT, K‐R and K‐Q. 5 × 10^6^ of HOS cells for expressing KDM2B WT, K‐R or K‐Q were subcutaneously injected into the hind limbs of mice. The mice injected with HOS cells which were transfected with empty vector were used as blank controls. Every five days, the tumour volume was measured.

### 2.15 Statistics

Data were presented as mean ± SD from three independent experiments. Statistical analyses were done by one‐way ANOVA in spss 19.0 software (Chicago, IL). *P* < 0.05 was considered to indicate the significant result.

## 3 RESULTS

### 3.1 KDM2B is acetylated in osteosarcoma cancer cells and Tip60 induces KDM2B acetylation

**Figure 1:**
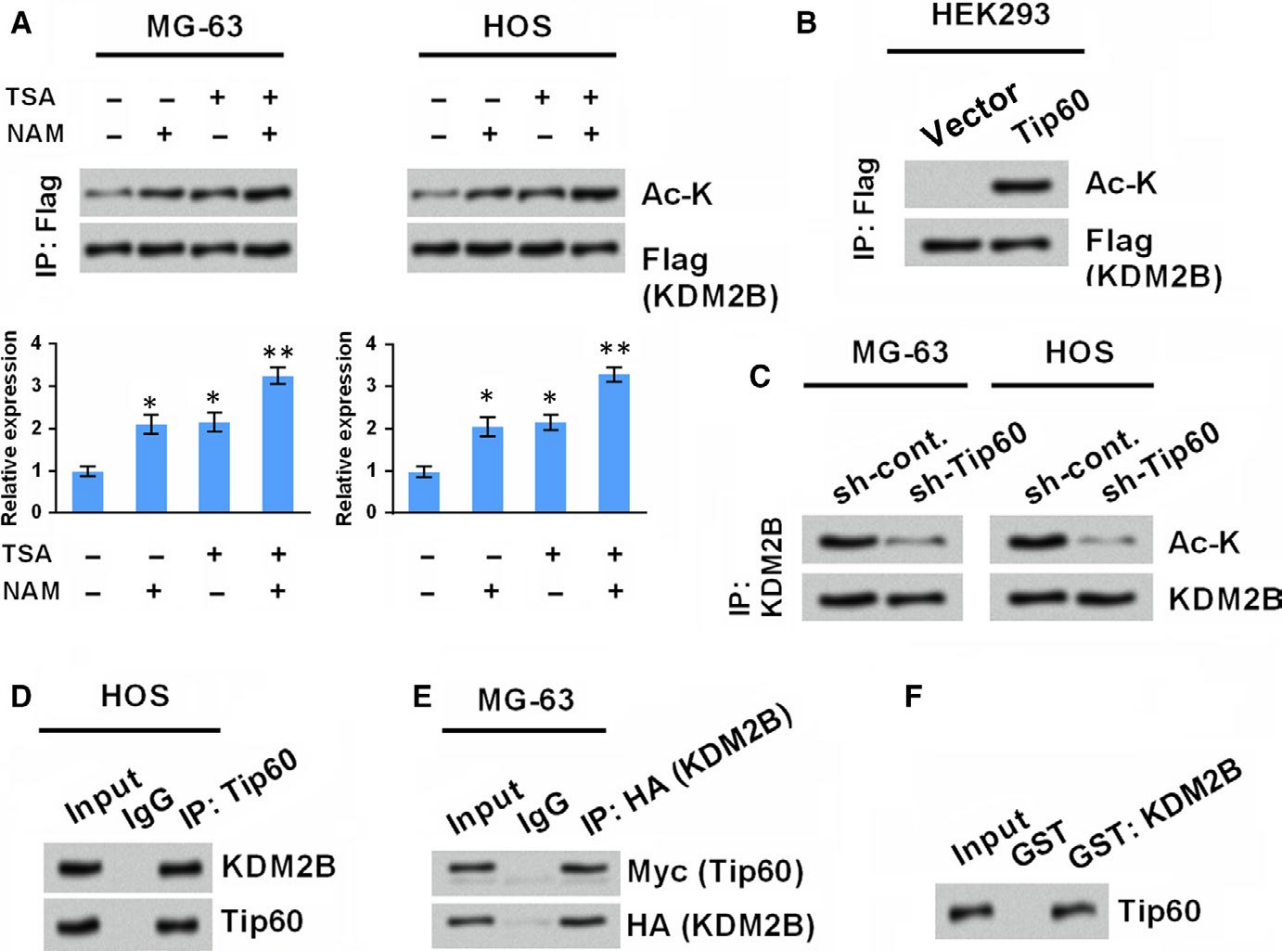
KDM2B is acetylated by Tip60. (A) KDM2B is acetylated in osteosarcoma cancer cell lines (MG‐63 and HOS), and inhibitors of HDAC, that is, Trichostatin A (TSA) and Nicotinamide (NAM) promote the acetylation. MG‐63 and HOS cells were transduced with lentiviral Flag‐KDM2B and thus treated by TSA and/or NAM for 24 h. Cell lysates were immunoprecipitated with anti‐Flag antibody and immunoblotted with antibodies specific for acetyl‐lysine (Ac‐K) and Flag. (B) KDM2B is acetylated by Tip60. Flag‐KDM2B was transfected into HEK293 cells alone or in combination with Tip60 expression vector. Immunoprecipitation was performed with anti‐Flag antibody, and immunoblotting was carried out by using anti‐Ac‐K and anti‐Flag antibodies. (C) Tip60 induces the acetylation of endogenous KDM2B. MG‐63 and HOS cells were transfected with shRNAs specific against Tip60 (sh‐Tip60). A lentiviral vector inserting with non‐targeting sequences was transfected as a negative control (sh‐cont.). Endogenous KDM2B proteins were immunoprecipitated with anti‐KDM2B antibody, followed by immunoblotting with anti‐Ac‐K and anti‐KDM2B antibodies. (D) HOS cell lysates were immunoprecipitated with anti‐Tip60 antibody followed by immunoprecipitated with antibodies against KDM2B and Tip60. (E) MG‐63 cell lysates were immunoprecipitated with anti‐HA antibody followed by immunoprecipitated with antibodies against Myc and Flag to detect Tip60. (F) Glutathione S‐transferase (GST) pull‐down was performed to test the association between GST‐KDM2B and human HA‐tagged Tip60. *P < 0.05, **P < 0.01 vs the non‐treated control group

Initially, we tested whether KDM2B underwent acetylation in osteosarcoma cancer cells by performing co‐immunoprecipitation (CoIP). To this end, two osteosarcoma cancer cell lines, MG‐63 and HOS, were transfected with Flag‐tagged KDM2B vector and the proteins extracted from the transfected cells were immunoprecipitated with anti‐Flag. It was observed that KDM2B acetylation occurred in both MG‐63 and HOS cells (Figure 1A). Treating MG‐63 and HOS cells with HDAC inhibitors, TSA and NAM, further enhanced the acetylation of KDM2B (*P* < 0.05), and combination of TSA and NAM acquired the highest level of KDM2B acetylation than use TSA or NAM separately (*P* < 0.01, Figure 1A). These findings suggested that KDM2B was acetylated in MG‐63 and HOS cells and diverse kinds of HDAC inhibitors can participate in the acetylation of KDM2B.

The enzyme responsible for the acetylation of KDM2B was further studied. HEK293, a cell line with a reliable propensity for transfection was transfected with a Tip60 expression vector in combination with Flag‐tagged KDM2B vector. CoIP results in Figure 1B showed that, Tip60 could promote the acetylation of KDM2B, indicating KDM2B acetylation might be mediated by Tip60. This phenomenon was further confirmed in MG‐63 and HOS cells. As shown in Figure 1C, silence of Tip60 by the specific shRNAs reduced the endogenous level of KDM2B acetylation.

Next, the interaction between KDM2B and Tip60 in MG‐63 and HOS cells was studied. Immunoprecipitation of endogenous Tip60 protein was performed with the specific antibody, and the complex can be co‐precipitated with KDM2B (Figure 1D). Similarly, exogenous Tip60 was also co‐precipitated with KDM2B (Figure 1E). And also, the interaction between KDM2B and Tip60 was verified by GST (Figure 1F). Taken together, the data demonstrate that KDM2B acetylation may be directly mediated by Tip60.

### 3.2 Tip60 directly acetylates KDM2B at K758

**Figure 2:**
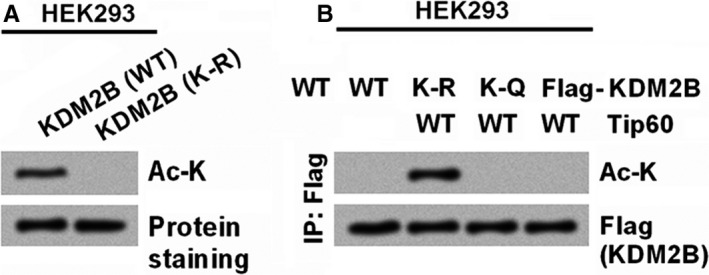
KDM2B is acetylated by Tip60 at K758. (A) Acetylation of the KDM2B by Tip60. HEK293 cells were transfected with vectors expressing Flag‐Tip60, followed by immunoprecipitation with anti‐Flag antibody. The precipitates were incubated with recombinant KDM2B (WT or K‐R). Acetylation of KDM2B was determined by immunoblotting with anti‐Ac‐K antibodies. (B) Lysine 758 of KDM2B is the Tip60 acetylation site. Flag‐tagged WT and mutants (K‐R and K‐Q) of KDM2B were co‐expressed with WT‐Tip60 in HEK293 cells. Acetylation of KDM2B was evaluated by immunoprecipitation with anti‐Flag antibodies and immunoblotting with anti‐Ac‐K antibody

As data released by an online database (https://www.phosphosite.org/homeAction), the acetylation event in KDM2B usually occurs at K758. Thereby, we next detected if Tip60 acetylated the KDM2B at K758. Recombinant full‐length of KDM2B proteins were generated from *Escherichia coli*. To make a K‐R mutant of KDM2B, the K758 in KDM2B was substituted with R. CoIP results in Figure 2A revealed that, the KDM2B was acetylated when incubated with the Tip60 immunoprecipitated in HEK293 cells. By contrast, the K‐R mutant, KDM2B, was resistant to Tip60‐mediated acetylation (Figure 2A). This result was consistent with direct acetylation of KDM2B by Tip60. When the K758 of KDM2B was replaced by R, the mutant efficiently resisted Tip60‐mediated acetylation (Figure 2B). Similarly, when the K758 of KDM2B was replaced with glutamine (Q), the resulting K‐Q mutant KDM2B also resisted Tip60‐mediated acetylation (Figure 2B). These results confirm K758 is the Tip60 acetylation site in KDM2B proteins.

### 3.3 Acetylation of KDM2B disrupts its demethylation activity and the transcription of target genes

**Figure 3:**
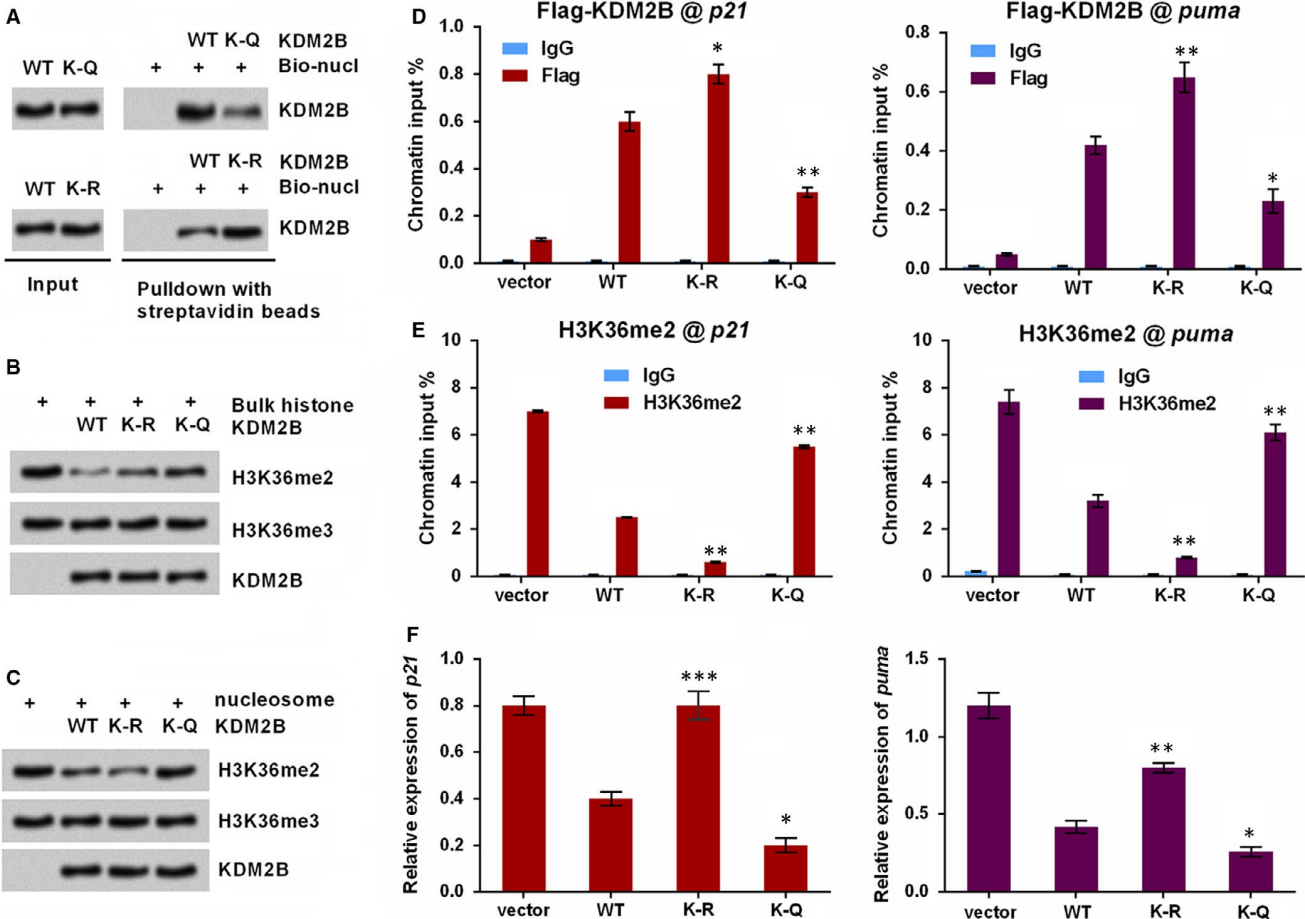
Acetylation of KDM2B disrupts its demethylation activity and the transcription of target genes. (A) KDM2B acetylation attenuates its association with nucleosomes. The nucleosomes prepared by recombinant core histones and biotin‐labelled DNA (Bio‐nucl) were incubated with recombinant KDM2B proteins (WT, K‐Q, K‐R). Nucleosomes were then isolated and associated proteins were immunoblotted with anti‐KDM2B antibody. (B) The demethylation activity of KDM2B is unaffected in bulk histones. After incubation with recombinant KDM2B (WT, K‐R or K‐Q), the bulk histones were immunoblotted with the antibodies against H3K36me2, H3K36me3 and KDM2B. (C) The demethylation activity of KDM2B is influenced in nucleosomal substrates. Mononucleosomes were prepared and incubated with recombinant KDM2B (WT, K‐R or K‐Q). Histone demethylation was verified by immunoblotting. (D) Binding of KDM2B proteins (WT, K‐R or K‐Q) to the *p21* and *puma* promoters was evaluated by ChIP analysis with anti‐Flag antibody. IgG was used as a non‐specific antibody control. (E) H3K36me2 levels at the *p21* and *puma* promoters were determined by ChIP. (F) qRT‐PCR for detecting the expression levels of *p21* and *puma*. *P < 0.05, **P < 0.01, ***P < 0.001 vs WT group

In order to demethylate histone tails in chromatin, KDM2B must associate with nucleosomes. Thus, we nest investigated whether the acetylation of KDM2B impacted its binding effects on nucleosomes. To this end, recombinant WT, K‐R and K‐Q of KDM2B proteins were respectively incubated with mononucleosomes which were reconstituted with recombinant core histones and biotin‐tagged DNA fragments. As shown in Figure 3A, the association of KDM2B with nucleosomes was detected. However, such association was clearly repressed when K758 of KDM2B was mutated by Q and was increased when mutated by R. This finding suggested that, acetylation of KDM2B at K758 may decrease the capacity of KDM2B in binding with nucleosomes.

Considering KDM2B acetylation impairs its association with nucleosomes, we figure out that KDM2B acetylation can also impact its capacity of demethylating nucleosomal substrates. Herein, we found that recombinant KDM2B protein or its mutated types (K‐R and K‐Q) all remarkably decreased H3K36me2 levels when incubated with bulk histones (Figure 3B), indicating that the enzymatic activity of KDM2B on free histones could not be impacted by acetylation at K758. However, K‐Q mutant KDM2B failed to demethylate H3K36me2 when incubated with mononucleosomes (Figure 3C), suggesting KDM2B acetylation at K758 diminished its demethylation activity towards nucleosomal substrates. More interestingly, these data identified K‐R as an acetylation‐resistant mutant of KDM2B, and K‐Q as an acetylation‐mimetic mutant of KDM2B.

Chromatin immunoprecipitation was carried out to test the effects of KDM2B acetylation on the transcription of its target genes, like *p21* and *puma*. *p21* and *puma* are the two key genes in regulating tumour cells growth as well. Figure 3D showed that the recombinant KDM2B could bind with the promoters of *p21* and *puma*, and the binding effects were enhanced when the K758 of KDM2B was mutated by R. However, the enrichment of KDM2B enhanced by the promoter of *p21* and *puma* was declined when K758 was mutated by Q (*P* < 0.01 or *P* < 0.05, Figure 3D). Contrary trends were observed in Figure 3E, in which the declined enrichment of H3K36me2 was observed in K‐R mutant KDM2B group (*P* < 0.01). K‐Q mutant KDM2B group failed to decrease H3K36me2 level at these genomic loci (*P* < 0.01, Figure 3E). In addition, qRT‐PCR data in Figure 3F showed that K‐R mutant KDM2B group increased the mRNA levels of *p21* and *puma* (*P* < 0.001 and *P* < 0.01), whereas K‐R mutant KDM2B group declined the expression of these two genes as compared to WT KDM2B group (*P* < 0.05). This phenomenon was consistent with the above‐mentioned results, collectively suggested that the acetylation of KDM2B diminished its ability to bind with the promoter of target genes and demethylate H3K36.

### 3.4 The K758 acetylation is critical to KDM2B‐induced carcinogenesis

**Figure 4:**
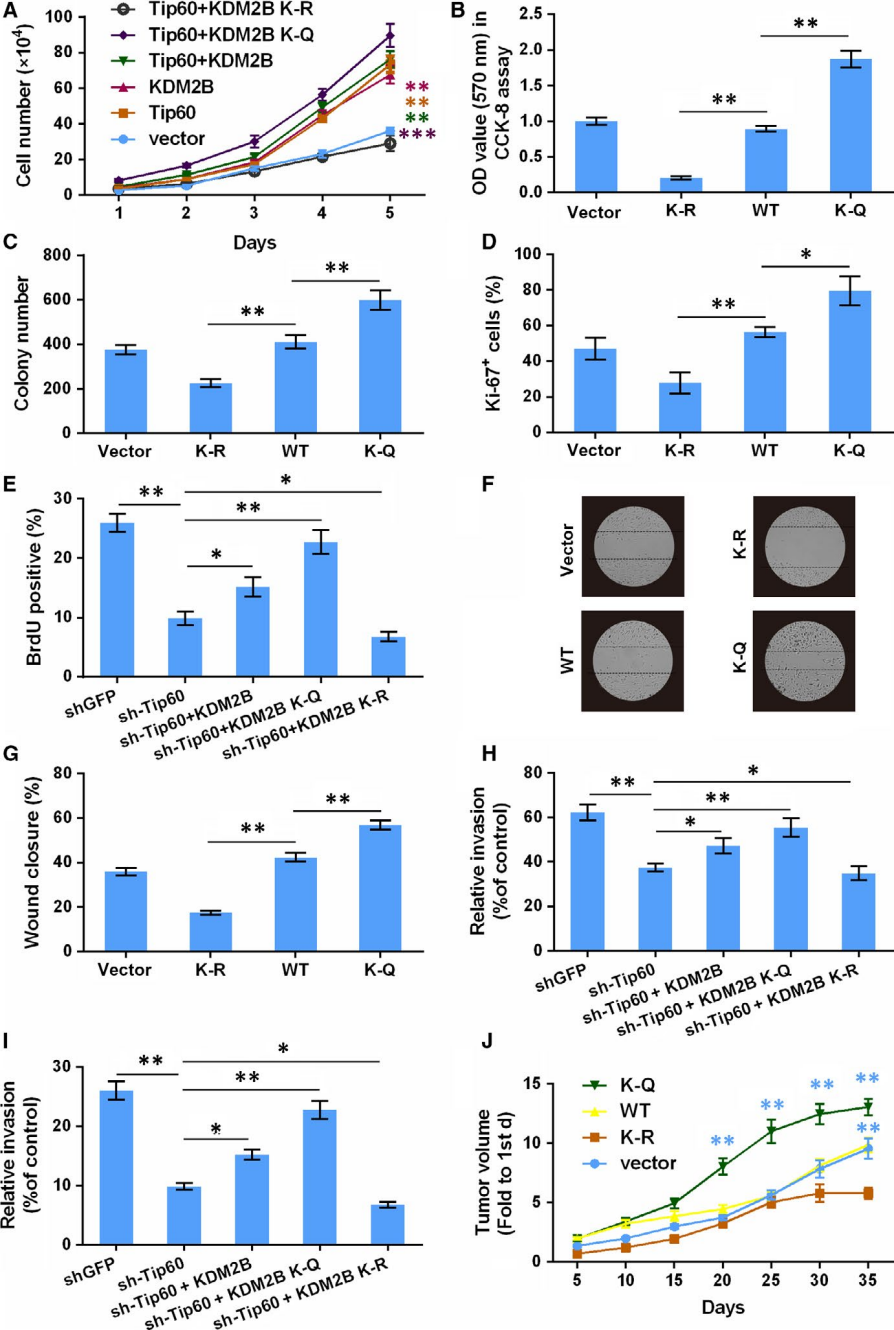
Acetylation of KDM2B promotes tumourigenesis. After the indicated transfection, following parameters were measured. (A) Growth curve of HOS cells; (B) the viability of MG‐63 cells tested by CCK‐8 assay; (C) colony formation capacity of HOS cells; (D) HOS cells proliferation tested by Ki‐67 staining; (E) MG‐63 cells proliferation tested by BrdU incorporation assay; (F,G) wound healing of HOS cells; (H,I) invasion of HOS and MG‐63 cells by Transwell assay. (J) HOS cells transfected with HA‐tagged KDM2B‐expressing plasmids were transferred into NOD/SCID mice. The tumour formation capabilities were tested by in vivo tumour formation assay. *P < 0.05, **P < 0.01, ***P < 0.001 vs the indicated group (indicated by different colours or horizontal lines)

Since KDM2B acetylation at K758 has the ability to mediate the transcription of *p21* and *puma*, two key genes in modulating tumour cells growth and apoptosis, we next investigated whether KDM2B acetylation was implicated in the carcinogenesis of osteosarcoma. First, we observed that overexpression of Tip60 and KDM2B promoted HOS cells viability, while KDM2B^K758Q^ enhanced this effect and KDM2B^K758R^ retarded it (Figure 4A). The effects of KDM2B^K758Q^ and KDM2B^K758R^ on tumour cells viability was also verified in MG‐63 cells by using CCK‐8 assay (Figure 4B). Results showed that, the viability of MG‐63 cells was reduced by KDM2B^K758R^ while was increased by KDM2B^K758Q^ (*P* < 0.01). Same trends were observed in the colony formation (*P* < 0.01, Figure 4C) and proliferation of HOS cells (*P* < 0.01 and *P* < 0.05, Figure 4D). Moreover, silence of Tip60 alone or in combination with overexpression of acetylation‐resistant KDM2B (KDM2B^K758R^) decreased MG‐63 cells proliferation, which was tested by BrdU assay. However, the overexpression of WT or acetylation‐mimetic KDM2B (KDM2B^K758Q^) rescued it (Figure 4E). Taken together, these results indicated that acetylation of KDM2B promoted cancer cells growth.

Further, we analysed whether KDM2B acetylation promoted the migration of cancer cells. First, WT or mutation KDM2B expression plasmids were transfected into HOS and MG‐63 cells. The results of wound healing assay indicated that acetylation‐mimetic KDM2B (KDM2B^K758Q^) obviously promoted cell migration, but acetylation‐resistant KDM2B (KDM2B^K758R^) has an opposite effect (*P* < 0.01, Figure 4F,G). Next, we observed that knockdown of Tip60 remarkably weakened HOS and MG‐63 cells invasion in Transwell assays. The overexpression of WT or KDM2B^K758Q^, rather than overexpression of KDM2B^K758R^ rescued invasion capability (Figure 4H,I).

Finally, we observed that KDM2B^K758Q^, but not the acetylation‐resistant form, has an important role in promoting tumour formation in mice (Figure 4J). Taken all the above results together, acetylation of KDM2B enhanced its transcriptional activity and thereby promotes tumourigenesis.

### 3.5 The tumour‐promoting effects of KDM2B acetylation are associated with p53

**Figure 5:**
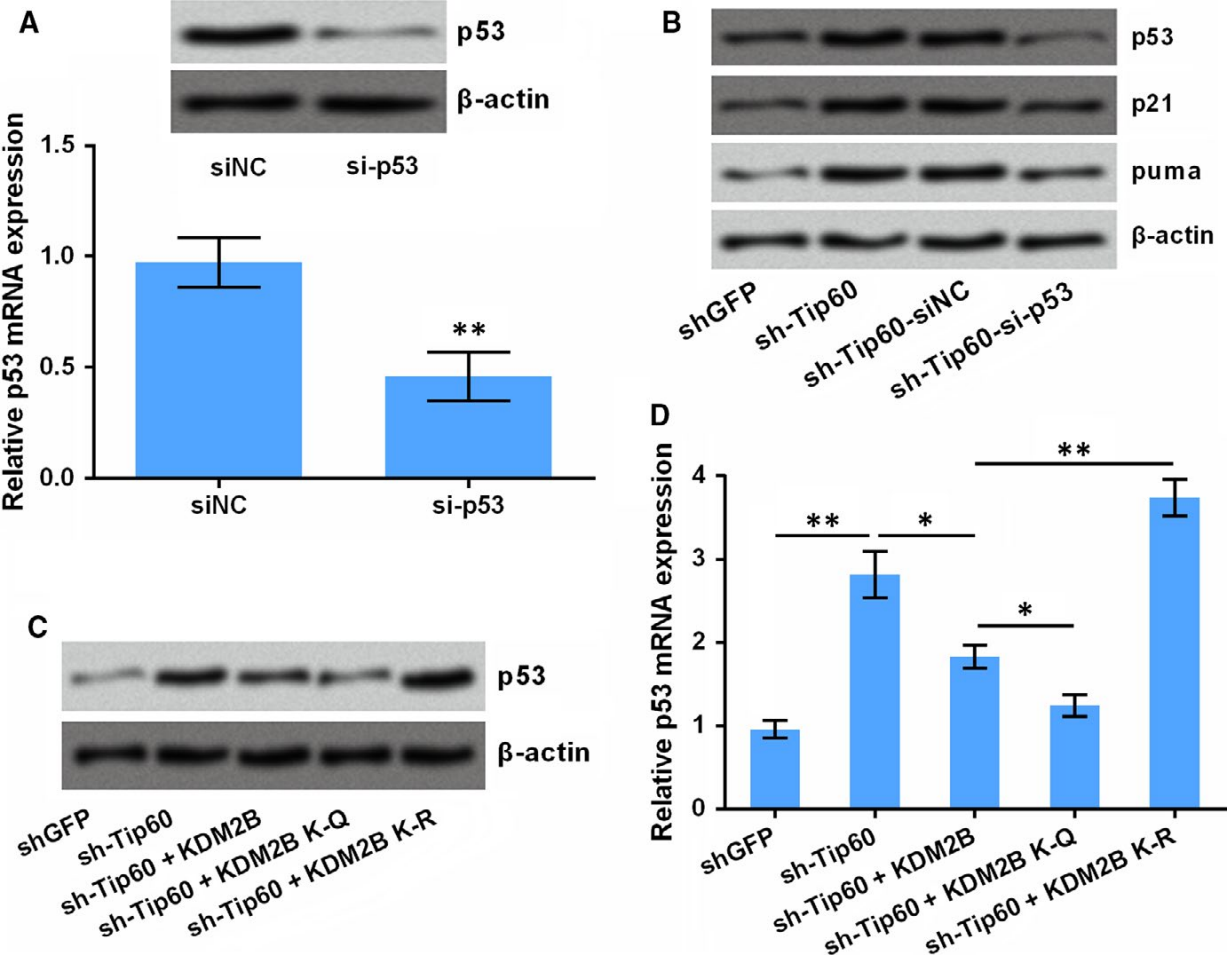
The tumour‐promoting effects of KDM2B acetylation are associated with p53. (A) HOS cells were transfected with siRNA specific for p53 (si‐p53) and its negative control (siNC). The protein and mRNA levels of p53 were detected by immunoblotting and qRt‐PCR respectively. (B) HOS cells were transfected as indicated, the protein levels of p53, p21 and puma was tested by immunoblotting. (C,D) HOS cells were transfected as indicated, the protein levels of p53 was tested by immunoblotting. *P < 0.05, **P < 0.01 vs the indicated group (indicated by horizontal lines)

It is well‐known that p53 regulates the acetyltranferase Tip60 in cancer.^22^ Thus, we have tested the possible interaction between p53 and Tip60 in the presence of KDM2B^K758Q^ or KDM2B^K758R^. To this end, the expression of p53 in HOS cells was silenced by siRNA‐mediated transfection. Data in Figure 5A showed that, both protein and mRNA levels of p53 were declined by siRNA specific for p53 (si‐p53) (*P* < 0.01). More interestingly, silence of Tip60 increased the protein expression of p53, p21 and puma, while silence of p53 flattened the increase of p21 and puma made by Tip60 silence (Figure 5B). These results indicated the negative regulatory effects of Tip60 on p53, p21 and puma expression. Further results demonstrated that, transfection of cells with KDM2B expression vector could also decrease p53 expression (*P* < 0.05). Besides that, p53 expression was further decreased by KDM2B^K758Q^ while was recovered by KDM2B^K758R^ (*P* < 0.05 and *P* < 0.01, Figure 5C,D). This result indicated that the declined expression of p53 may contribute in the tumour‐promoting effects of KDM2B acetylation.

## 4 DISCUSSION

Overexpression of KDM2B was frequently occurred in various human solid tumours, like triple‐negative breast cancer,^23^ pancreatic ductal adenocarcinoma^14^ and prostate cancer.^24^ Besides, the high levels of KDM2B are associated with tumour aggressiveness, recurrence and adverse prognosis through controlling of tumour cells growth, metastasis, apoptosis and drug resistance.^25^ So far, the oncogenic role of KDM2B in various types of cancers has been revealed. For the selected examples, KDM2B inhibited the growth, proliferation and metastasis of ovarian cancer,^13^ pancreatic cancer^14^ and bladder cancer.^26^ However, whether and how its activities might be modulated to facilitate tumour progression is still unclear. In the current study, we found that KDM2B was acetylated in osteosarcoma cancer cells. This modification occurred at K758 and catalysed by Tip60. More interestingly, in vitro and in vivo data confirmed the oncogenic effects of KDM2B acetylation which might be owned to the diminished ability to bind with target genes and demethylate H3K36. These findings collectively suggested Tip60‐mediated acetylation of KDM2B is a critical switch in modulating its oncogenic function in the onset and progression of human osteosarcoma.

Just as with the other proteins, KDM2B can be post‐translationally modified via various modification types, like phosphorylation, acetylation, ubiquitination, methylation and sumoylation. These modifications occur in diverse amino acid residues of KDM2B, which induce its covalent linkage to diverse functional groups,^27^ and thus influence its activity, function and specific responses to different treatments. In this study, acetylation of KDM2B was observed in human osteosarcoma cell lines (MG‐63 and HOS). Besides, by using site mutation technique, it was found that the acetylation site is K758. The high occurrence of KDM2B acetylation at K758 in osteosarcoma cells indicated that the oncogenic activities of KDM2B might depend on its acetylation at K758.

KDM2B is a member of epigenetic modifiers which is responsible for demethylating lys‐4 and lys‐36 of histone H3. In order to further reveal the impacts of acetylation on KDM2B's function, the enzymatic activity of KDM2B was detected. We found that the demethylation activity of KDM2B was not impacted towards free histones substrates, but was diminished in nucleosomal substrates. This result suggested that KDM2B acetylation disrupted its demethylation activity via inhibiting nucleosomes‐binding capacities without influencing the enzymatic activity. *p21* is a well‐known regulator of cell cycle progression, which has potent inhibitory effects on cyclin‐dependent kinase (CDK). *p21* was found to be frequently down‐regulated in osteosarcoma, and it exhibited inhibitory roles in the growth of this cancer.^28^, ^29^ *puma*, also known as *BBC3*, is a pro‐apoptotic gene. *puma* induces mitochondrial dysfunction and caspase activation, and thus mediates cell death signals.^30^ Because of that, *puma* has been considered as a promising drug target for cancer therapy.^31^, ^32^ The present work revealed that KDM2B transcriptionally reduced the expression of *p21* and *puma* via binding with the promoters of these two genes, indicating *p21* and *puma* are two downstream effectors of KDM2B. More interestingly, these effects were enhanced by acetylation‐resistant mutant of KDM2B, while were recovered by acetylation‐mimetic mutant of KDM2B. Thus, it seems that acetylation of KDM2B diminished its ability to bind with target genes and thus exhibited oncogenic effects. This hypothesis was further confirmed by performing in vitro and in vivo experiments, as the proliferation, colony formation, migration and invasion of tumour cells were all enhanced by acetylation‐resistant mutant of KDM2B.

Next, the present work attempted to reveal whether KDM2B was acetylated via a well‐known lysine acetyltransferase Tip60. The acetylation of KDM2B was found to be mediated by Tip60. Besides, KDM2B acetylation conferred its oncogenic functions possibly via a Tip60‐dependent manner. Despite previous studies have reported the acetyltransferase activity of Tip60 towards both histone and non‐histone proteins,^15^, ^16^ this study for the first provided in vitro evidence that Tip60 exhibited catalytic activity towards KDM2B acetylation.

Transcriptional factor p53 can be activated by a variety of genotoxic stress such as DNA damage, hypoxia and oxidative stress. In cancer, the activation of p53 can inhibit the growth of tumour cells by inducing cell cycle arrest, apoptosis or cellular senescence. Besides, in cancer, p53 regulates the acetyltranferase Tip60,^22^ and Tip60 is required for p53 activation.^33^ The previous findings indicated the close association between Tip60 and p53. In the current study, p53 expression was found to be up‐regulated when Tip60 was silenced by siRNA‐mediated transfection. Acetylation of KDM2B eliminated the effects of Tip60 silence on p53 expression. It seems that Tip60 regulates the p53 expression owing to the acetylation of KDM2B. Our findings also showed that the elevated expression of p21 and puma proteins made by Tip60 silence was repressed by p53 silence. The result suggested that, p53 was involved in the tumourigenesis promoting the effects of Tip60 through disturbing its regulation on p21 and puma expression.

In conclusion, this study demonstrates that KDM2B is acetylated in human osteosarcoma cells, and the acetylation is mediated by Tip60 and occurs in K758. Acetylation of KDM2B diminishes its association with nucleosomes, and thus increasing methylation of H3K36 at its target genes. Moreover, Tip60‐dependent acetylation of KDM2B enhanced its pro‐proliferating and pro‐metastatic effects on osteosarcoma cells and in vivo tumour growth.

## CONFLICT OF INTEREST

Authors declare that there is no conflict of interests.

## Data Availability

The datasets used and/or analysed during the current study are available from the corresponding author on reasonable request.
